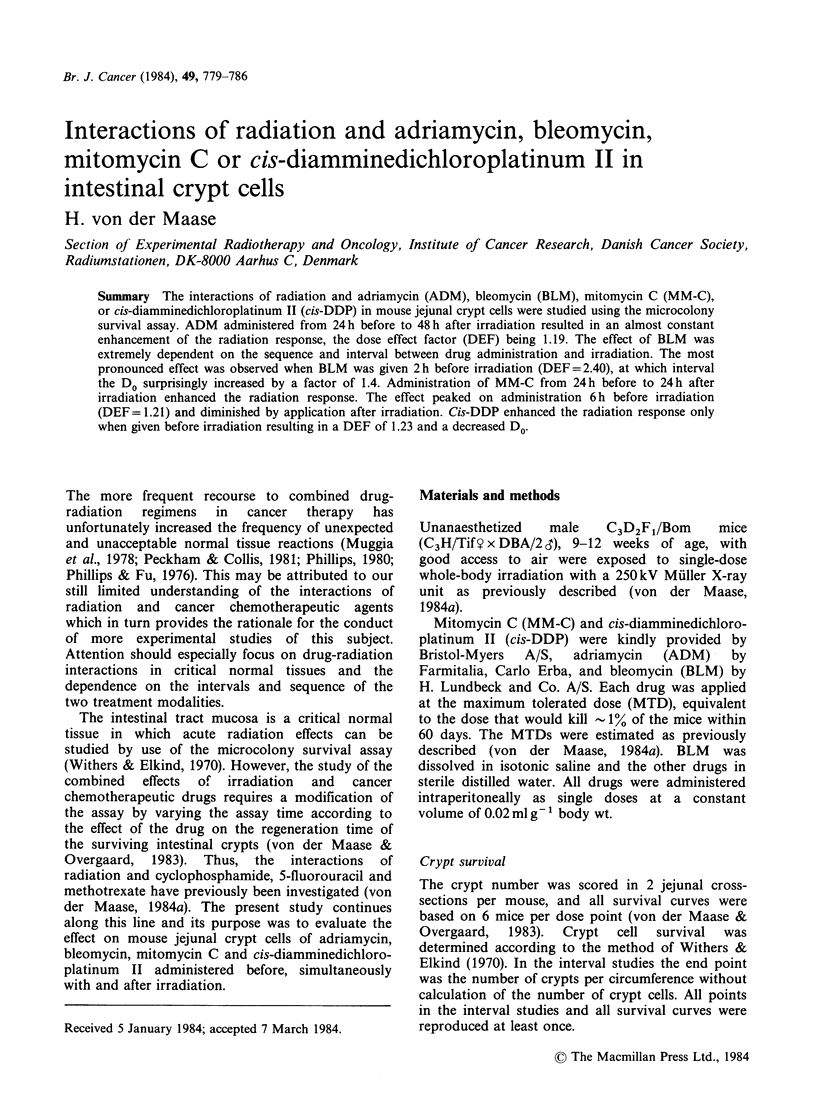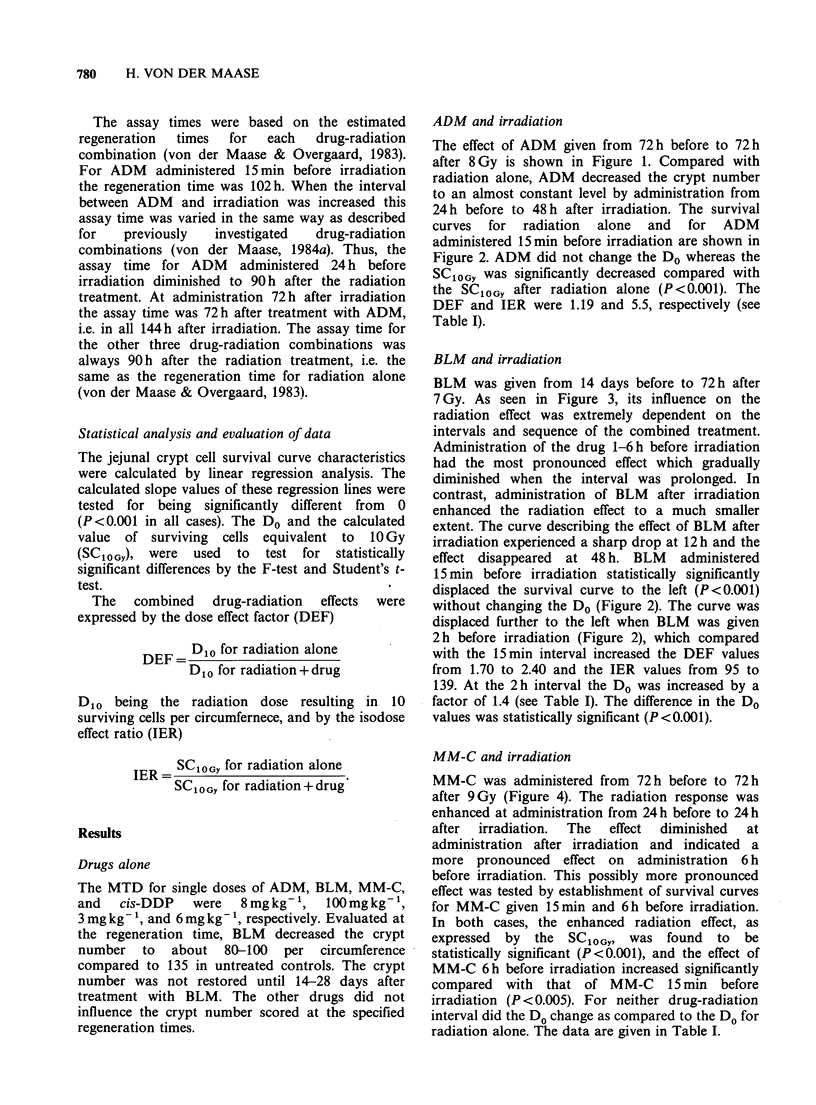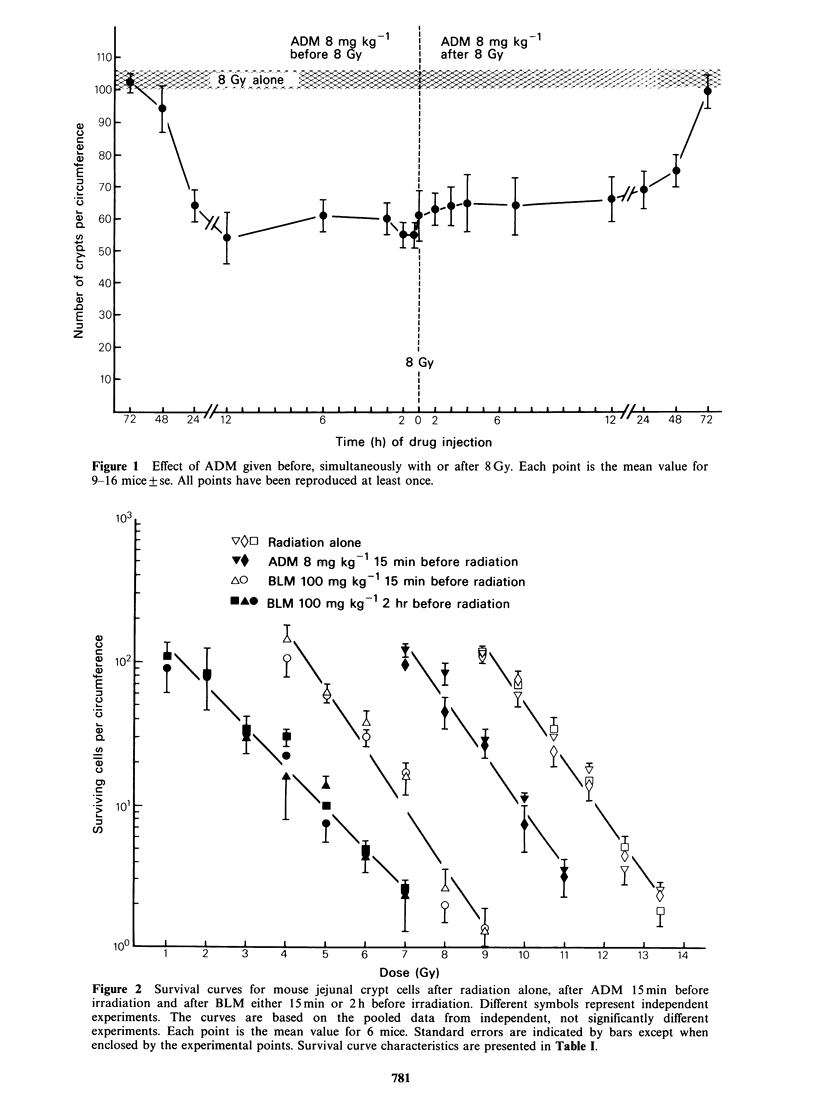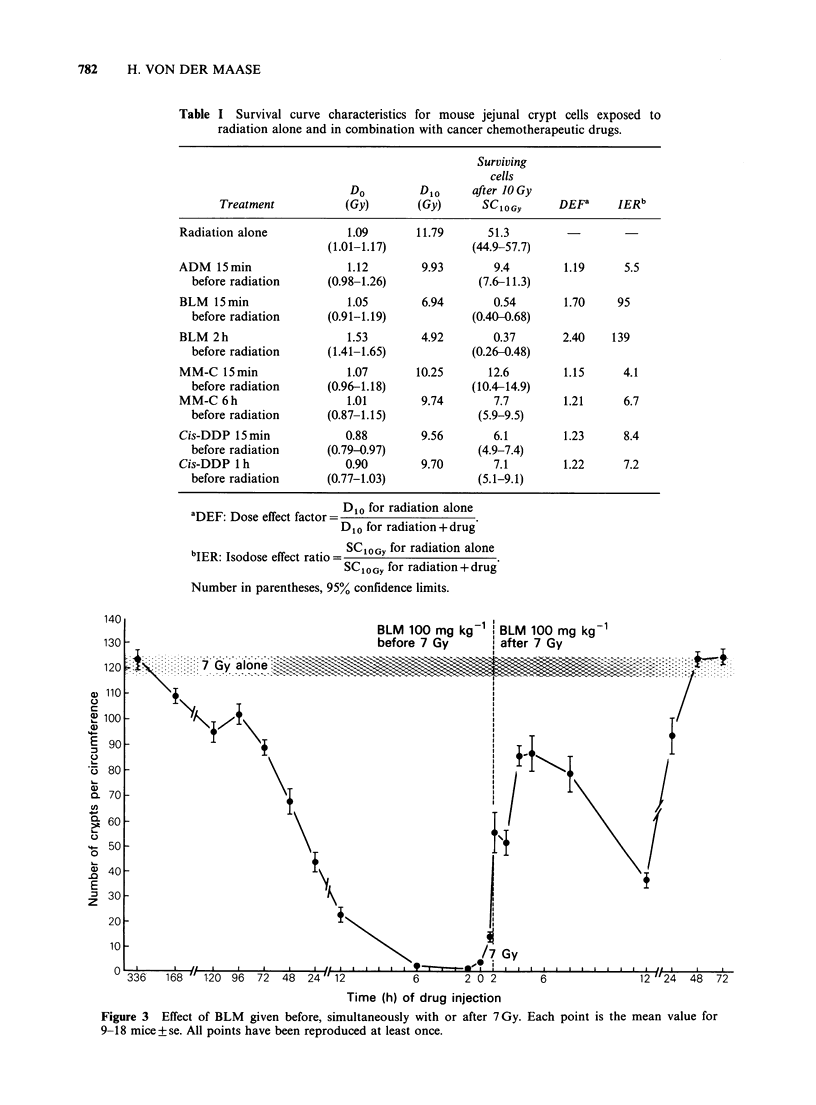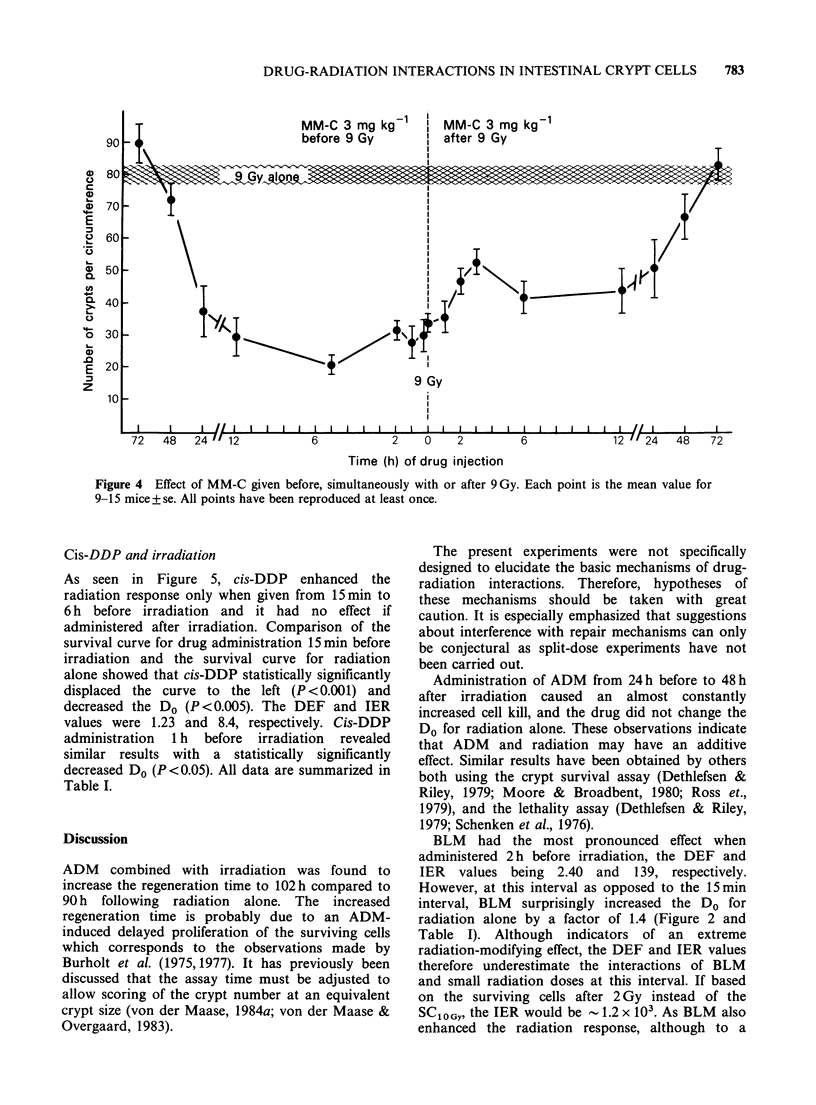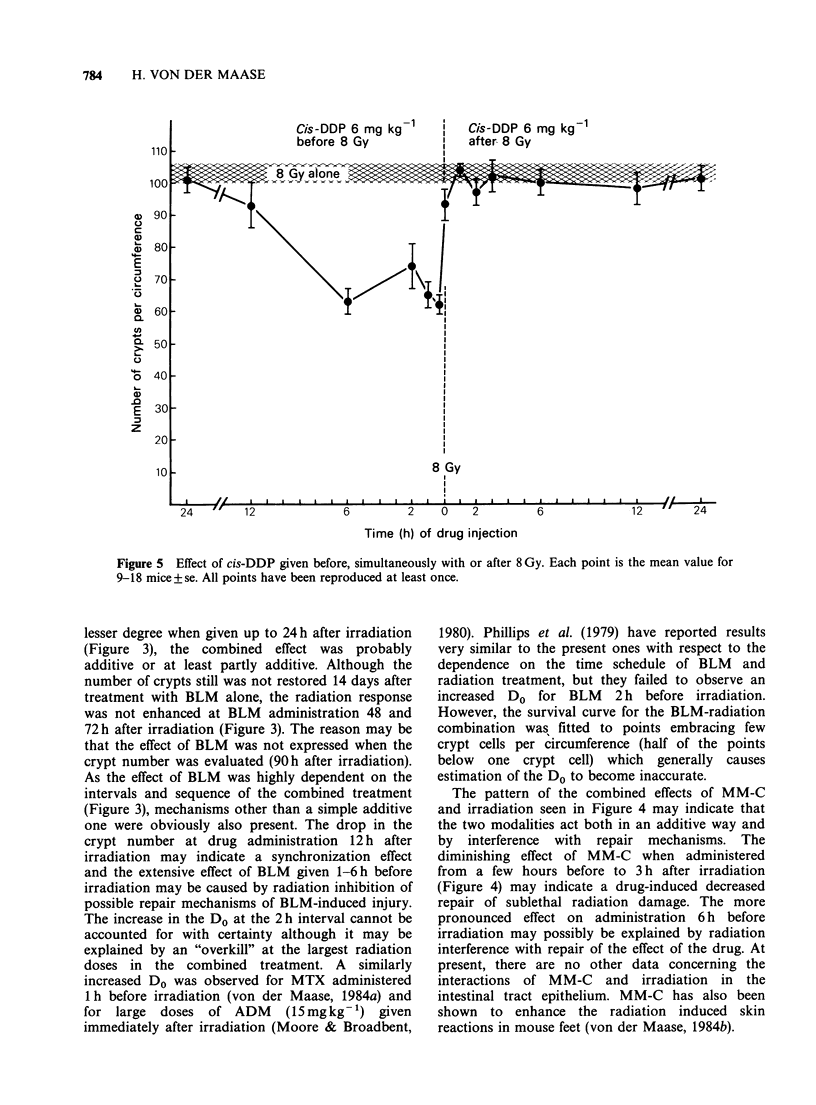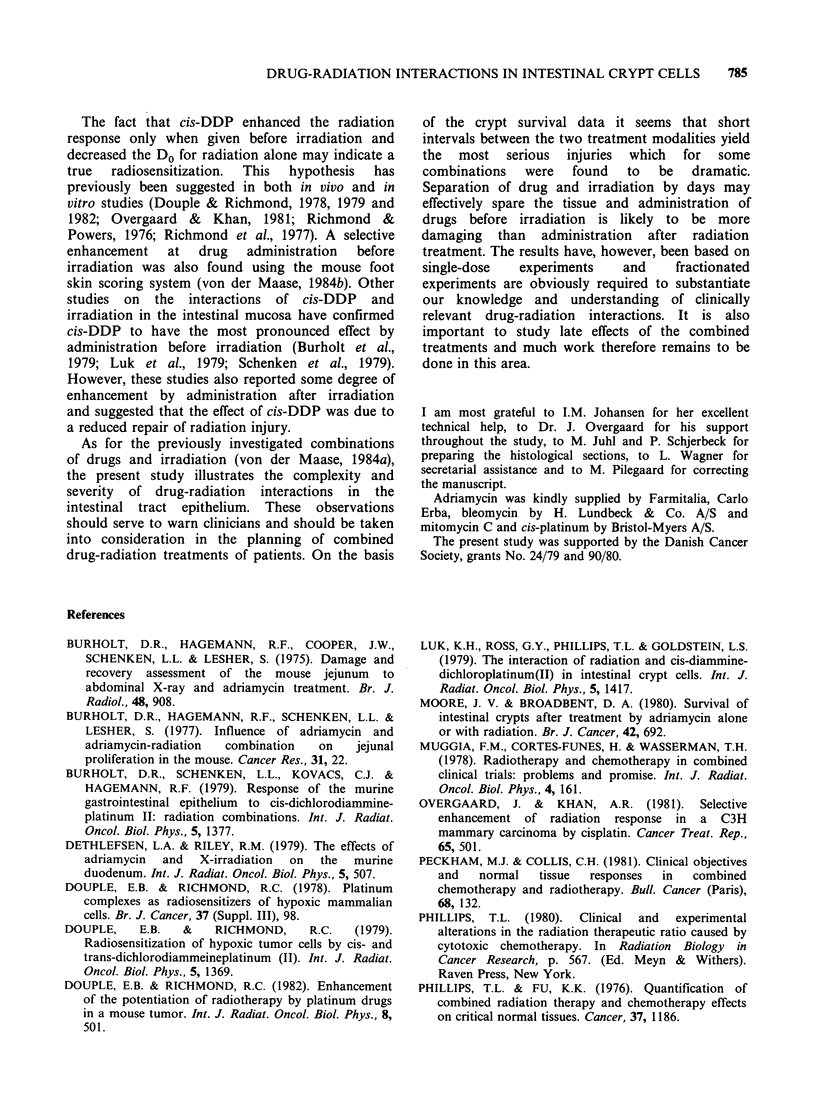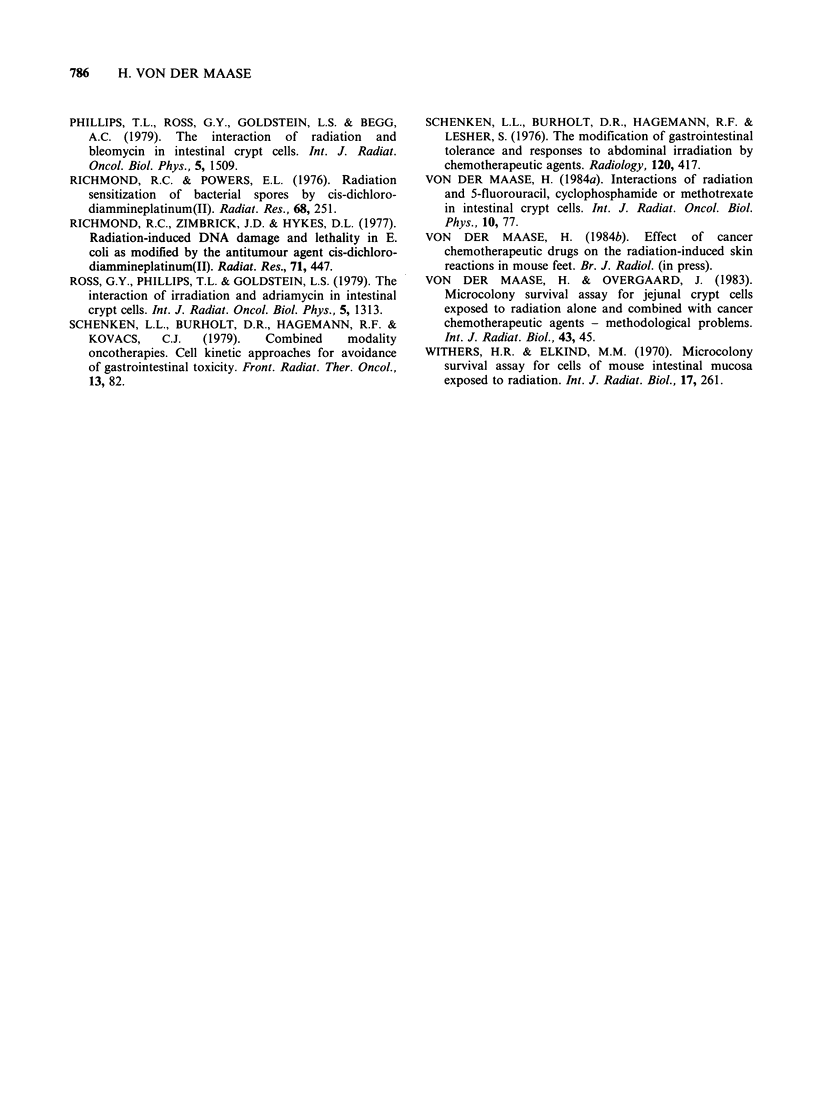# Interactions of radiation and adriamycin, bleomycin, mitomycin C or cis-diamminedichloroplatinum II in intestinal crypt cells.

**DOI:** 10.1038/bjc.1984.121

**Published:** 1984-06

**Authors:** H. von der Maase

## Abstract

The interactions of radiation and adriamycin (ADM), bleomycin (BLM), mitomycin C (MM-C), or cis-diamminedichloroplatinum II (cis-DDP) in mouse jejunal crypt cells were studied using the microcolony survival assay. ADM administered from 24 h before to 48 h after irradiation resulted in an almost constant enhancement of the radiation response, the dose effect factor (DEF) being 1.19. The effect of BLM was extremely dependent on the sequence and interval between drug administration and irradiation. The most pronounced effect was observed when BLM was given 2 h before irradiation (DEF = 2.40), at which interval the D0 surprisingly increased by a factor of 1.4. Administration of MM-C from 24 h before to 24 h after irradiation enhanced the radiation response. The effect peaked on administration 6 h before irradiation (DEF = 1.21) and diminished by application after irradiation. Cis-DDP enhanced the radiation response only when given before irradiation resulting in a DEF of 1.23 and a decreased D0.


*Br. J. Cancer* (1984), **49**, 779–786

### Interactions of radiation and adriamycin, bleomycin, mitomycin C or *cis*-diamminedichloroplatinum II in intestinal crypt cells

H. von der Maase

*Section of Experimental Radiotherapy and Oncology, Institute of Cancer Research, Danish Cancer Society, Radiumstationen, DK-8000 Aarhus C, Denmark*

**Summary** The interactions of radiation and adriamycin (ADM), bleomycin (BLM), mitomycin C (MM-C), or *cis*-diamminedichloroplatinum II (*cis*-DDP) in mouse jejunal crypt cells were studied using the microcolony survival assay. ADM administered from 24 h before to 48 h after irradiation resulted in an almost constant enhancement of the radiation response, the dose effect factor (DEF) being 1.19. The effect of BLM was extremely dependent on the sequence and interval between drug administration and irradiation. The most pronounced effect was observed when BLM was given 2 h before irradiation (DEF = 2.40), at which interval the $D_0$ surprisingly increased by a factor of 1.4. Administration of MM-C from 24 h before to 24 h after irradiation enhanced the radiation response. The effect peaked on administration 6 h before irradiation (DEF = 1.21) and diminished by application after irradiation. *Cis*-DDP enhanced the radiation response only when given before irradiation resulting in a DEF of 1.23 and a decreased $D_0$.

The more frequent recourse to combined drug-radiation regimens in cancer therapy has unfortunately increased the frequency of unexpected and unacceptable normal tissue reactions (Muggia *et al.*, 1978; Peckham & Collis, 1981; Phillips, 1980; Phillips & Fu, 1976). This may be attributed to our still limited understanding of the interactions of radiation and cancer chemotherapeutic agents which in turn provides the rationale for the conduct of more experimental studies of this subject. Attention should especially focus on drug-radiation interactions in critical normal tissues and the dependence on the intervals and sequence of the two treatment modalities.

The intestinal tract mucosa is a critical normal tissue in which acute radiation effects can be studied by use of the microcolony survival assay (Withers & Elkind, 1970). However, the study of the combined effects of irradiation and cancer chemotherapeutic drugs requires a modification of the assay by varying the assay time according to the effect of the drug on the regeneration time of the surviving intestinal crypts (von der Maase & Overgaard, 1983). Thus, the interactions of radiation and cyclophosphamide, 5-fluorouracil and methotrexate have previously been investigated (von der Maase, 1984*a*). The present study continues along this line and its purpose was to evaluate the effect on mouse jejunal crypt cells of adriamycin, bleomycin, mitomycin C and *cis*-diamminedichloroplatinum II administered before, simultaneously with and after irradiation.

Received 5 January 1984; accepted 7 March 1984.

### Materials and methods

Unanaesthetized male $C_3D_2F_1$/Bom mice ($C_3H$/Tif♀ × DBA/2♂), 9–12 weeks of age, with good access to air were exposed to single-dose whole-body irradiation with a 250 kV Müller X-ray unit as previously described (von der Maase, 1984*a*).

Mitomycin C (MM-C) and *cis*-diamminedichloroplatinum II (*cis*-DDP) were kindly provided by Bristol-Myers A/S, adriamycin (ADM) by Farmitalia, Carlo Erba, and bleomycin (BLM) by H. Lundbeck and Co. A/S. Each drug was applied at the maximum tolerated dose (MTD), equivalent to the dose that would kill ~1% of the mice within 60 days. The MTDs were estimated as previously described (von der Maase, 1984*a*). BLM was dissolved in isotonic saline and the other drugs in sterile distilled water. All drugs were administered intraperitoneally as single doses at a constant volume of 0.02 ml $g^{-1}$ body wt.

### *Crypt survival*

The crypt number was scored in 2 jejunal cross-sections per mouse, and all survival curves were based on 6 mice per dose point (von der Maase & Overgaard, 1983). Crypt cell survival was determined according to the method of Withers & Elkind (1970). In the interval studies the end point was the number of crypts per circumference without calculation of the number of crypt cells. All points in the interval studies and all survival curves were reproduced at least once.

© The Macmillan Press Ltd., 1984

The assay times were based on the estimated regeneration times for each drug-radiation combination (von der Maase & Overgaard, 1983). For ADM administered 15 min before irradiation the regeneration time was 102 h. When the interval between ADM and irradiation was increased this assay time was varied in the same way as described for previously investigated drug-radiation combinations (von der Maase, 1984*a*). Thus, the assay time for ADM administered 24 h before irradiation diminished to 90 h after the radiation treatment. At administration 72 h after irradiation the assay time was 72 h after treatment with ADM, i.e. in all 144 h after irradiation. The assay time for the other three drug-radiation combinations was always 90 h after the radiation treatment, i.e. the same as the regeneration time for radiation alone (von der Maase & Overgaard, 1983).

### *Statistical analysis and evaluation of data*

The jejunal crypt cell survival curve characteristics were calculated by linear regression analysis. The calculated slope values of these regression lines were tested for being significantly different from 0 ($P<0.001$ in all cases). The $D_0$ and the calculated value of surviving cells equivalent to 10 Gy ($SC_{10\,Gy}$), were used to test for statistically significant differences by the F-test and Student's *t*-test.

The combined drug-radiation effects were expressed by the dose effect factor (DEF)

$$\mathrm{DEF}=\frac{D_{10}\text{ for radiation alone}}{D_{10}\text{ for radiation}+\text{drug}}$$

$D_{10}$ being the radiation dose resulting in 10 surviving cells per circumfernece, and by the isodose effect ratio (IER)

$$\mathrm{IER}=\frac{SC_{10\,Gy}\text{ for radiation alone}}{SC_{10\,Gy}\text{ for radiation}+\text{drug}}.$$

### Results

### *Drugs alone*

The MTD for single doses of ADM, BLM, MM-C, and *cis*-DDP were 8 mg kg$^{-1}$, 100 mg kg$^{-1}$, 3 mg kg$^{-1}$, and 6 mg kg$^{-1}$, respectively. Evaluated at the regeneration time, BLM decreased the crypt number to about 80–100 per circumference compared to 135 in untreated controls. The crypt number was not restored until 14–28 days after treatment with BLM. The other drugs did not influence the crypt number scored at the specified regeneration times.

### *ADM and irradiation*

The effect of ADM given from 72 h before to 72 h after 8 Gy is shown in Figure 1. Compared with radiation alone, ADM decreased the crypt number to an almost constant level by administration from 24 h before to 48 h after irradiation. The survival curves for radiation alone and for ADM administered 15 min before irradiation are shown in Figure 2. ADM did not change the $D_0$ whereas the $SC_{10\,Gy}$ was significantly decreased compared with the $SC_{10\,Gy}$ after radiation alone ($P<0.001$). The DEF and IER were 1.19 and 5.5, respectively (see Table I).

### *BLM and irradiation*

BLM was given from 14 days before to 72 h after 7 Gy. As seen in Figure 3, its influence on the radiation effect was extremely dependent on the intervals and sequence of the combined treatment. Administration of the drug 1–6 h before irradiation had the most pronounced effect which gradually diminished when the interval was prolonged. In contrast, administration of BLM after irradiation enhanced the radiation effect to a much smaller extent. The curve describing the effect of BLM after irradiation experienced a sharp drop at 12 h and the effect disappeared at 48 h. BLM administered 15 min before irradiation statistically significantly displaced the survival curve to the left ($P<0.001$) without changing the $D_0$ (Figure 2). The curve was displaced further to the left when BLM was given 2 h before irradiation (Figure 2), which compared with the 15 min interval increased the DEF values from 1.70 to 2.40 and the IER values from 95 to 139. At the 2 h interval the $D_0$ was increased by a factor of 1.4 (see Table I). The difference in the $D_0$ values was statistically significant ($P<0.001$).

### *MM-C and irradiation*

MM-C was administered from 72 h before to 72 h after 9 Gy (Figure 4). The radiation response was enhanced at administration from 24 h before to 24 h after irradiation. The effect diminished at administration after irradiation and indicated a more pronounced effect on administration 6 h before irradiation. This possibly more pronounced effect was tested by establishment of survival curves for MM-C given 15 min and 6 h before irradiation. In both cases, the enhanced radiation effect, as expressed by the $SC_{10\,Gy}$, was found to be statistically significant ($P<0.001$), and the effect of MM-C 6 h before irradiation increased significantly compared with that of MM-C 15 min before irradiation ($P<0.005$). For neither drug-radiation interval did the $D_0$ change as compared to the $D_0$ for radiation alone. The data are given in Table I.

**Figure 1** Effect of ADM given before, simultaneously with or after 8 Gy. Each point is the mean value for 9–16 mice ± se. All points have been reproduced at least once.

**Figure 2** Survival curves for mouse jejunal crypt cells after radiation alone, after ADM 15 min before irradiation and after BLM either 15 min or 2 h before irradiation. Different symbols represent independent experiments. The curves are based on the pooled data from independent, not significantly different experiments. Each point is the mean value for 6 mice. Standard errors are indicated by bars except when enclosed by the experimental points. Survival curve characteristics are presented in **Table I**.

**Table I** Survival curve characteristics for mouse jejunal crypt cells exposed to radiation alone and in combination with cancer chemotherapeutic drugs.

| *Treatment* | $D_0$ *(Gy)* | $D_{10}$ *(Gy)* | *Surviving cells after 10 Gy* $SC_{10Gy}$ | *DEF*[a] | *IER*[b] |
|---|---|---|---|---|---|
| Radiation alone | 1.09 (1.01–1.17) | 11.79 | 51.3 (44.9–57.7) | — | — |
| ADM 15 min before radiation | 1.12 (0.98–1.26) | 9.93 | 9.4 (7.6–11.3) | 1.19 | 5.5 |
| BLM 15 min before radiation | 1.05 (0.91–1.19) | 6.94 | 0.54 (0.40–0.68) | 1.70 | 95 |
| BLM 2 h before radiation | 1.53 (1.41–1.65) | 4.92 | 0.37 (0.26–0.48) | 2.40 | 139 |
| MM-C 15 min before radiation | 1.07 (0.96–1.18) | 10.25 | 12.6 (10.4–14.9) | 1.15 | 4.1 |
| MM-C 6 h before radiation | 1.01 (0.87–1.15) | 9.74 | 7.7 (5.9–9.5) | 1.21 | 6.7 |
| *Cis*-DDP 15 min before radiation | 0.88 (0.79–0.97) | 9.56 | 6.1 (4.9–7.4) | 1.23 | 8.4 |
| *Cis*-DDP 1 h before radiation | 0.90 (0.77–1.03) | 9.70 | 7.1 (5.1–9.1) | 1.22 | 7.2 |

[a]DEF: Dose effect factor $= \frac{D_{10} \text{ for radiation alone}}{D_{10} \text{ for radiation} + \text{drug}}$.

[b]IER: Isodose effect ratio $= \frac{SC_{10Gy} \text{ for radiation alone}}{SC_{10Gy} \text{ for radiation} + \text{drug}}$.

Number in parentheses, 95% confidence limits.

**Figure 3** Effect of BLM given before, simultaneously with or after 7 Gy. Each point is the mean value for 9–18 mice ± se. All points have been reproduced at least once.

**Figure 4** Effect of MM-C given before, simultaneously with or after 9 Gy. Each point is the mean value for 9–15 mice ± se. All points have been reproduced at least once.

### *Cis-DDP and irradiation*

As seen in Figure 5, *cis*-DDP enhanced the radiation response only when given from 15 min to 6 h before irradiation and it had no effect if administered after irradiation. Comparison of the survival curve for drug administration 15 min before irradiation and the survival curve for radiation alone showed that *cis*-DDP statistically significantly displaced the curve to the left ($P<0.001$) and decreased the $D_0$ ($P<0.005$). The DEF and IER values were 1.23 and 8.4, respectively. *Cis*-DDP administration 1 h before irradiation revealed similar results with a statistically significantly decreased $D_0$ ($P<0.05$). All data are summarized in Table I.

### Discussion

ADM combined with irradiation was found to increase the regeneration time to 102 h compared to 90 h following radiation alone. The increased regeneration time is probably due to an ADM-induced delayed proliferation of the surviving cells which corresponds to the observations made by Burholt *et al.* (1975, 1977). It has previously been discussed that the assay time must be adjusted to allow scoring of the crypt number at an equivalent crypt size (von der Maase, 1984*a*; von der Maase & Overgaard, 1983).

The present experiments were not specifically designed to elucidate the basic mechanisms of drug-radiation interactions. Therefore, hypotheses of these mechanisms should be taken with great caution. It is especially emphasized that suggestions about interference with repair mechanisms can only be conjectural as split-dose experiments have not been carried out.

Administration of ADM from 24 h before to 48 h after irradiation caused an almost constantly increased cell kill, and the drug did not change the $D_0$ for radiation alone. These observations indicate that ADM and radiation may have an additive effect. Similar results have been obtained by others both using the crypt survival assay (Dethlefsen & Riley, 1979; Moore & Broadbent, 1980; Ross *et.*, 1979), and the lethality assay (Dethlefsen & Riley, 1979; Schenken *et al.*, 1976).

BLM had the most pronounced effect when administered 2 h before irradiation, the DEF and IER values being 2.40 and 139, respectively. However, at this interval as opposed to the 15 min interval, BLM surprisingly increased the $D_0$ for radiation alone by a factor of 1.4 (Figure 2 and Table I). Although indicators of an extreme radiation-modifying effect, the DEF and IER values therefore underestimate the interactions of BLM and small radiation doses at this interval. If based on the surviving cells after 2 Gy instead of the $SC_{10\,Gy}$, the IER would be $\sim 1.2\times10^3$. As BLM also enhanced the radiation response, although to a

**Figure 5** Effect of *cis*-DDP given before, simultaneously with or after 8 Gy. Each point is the mean value for 9–18 mice ± se. All points have been reproduced at least once.

lesser degree when given up to 24 h after irradiation (Figure 3), the combined effect was probably additive or at least partly additive. Although the number of crypts still was not restored 14 days after treatment with BLM alone, the radiation response was not enhanced at BLM administration 48 and 72 h after irradiation (Figure 3). The reason may be that the effect of BLM was not expressed when the crypt number was evaluated (90 h after irradiation). As the effect of BLM was highly dependent on the intervals and sequence of the combined treatment (Figure 3), mechanisms other than a simple additive one were obviously also present. The drop in the crypt number at drug administration 12 h after irradiation may indicate a synchronization effect and the extensive effect of BLM given 1–6 h before irradiation may be caused by radiation inhibition of possible repair mechanisms of BLM-induced injury. The increase in the $D_0$ at the 2 h interval cannot be accounted for with certainty although it may be explained by an "overkill" at the largest radiation doses in the combined treatment. A similarly increased $D_0$ was observed for MTX administered 1 h before irradiation (von der Maase, 1984*a*) and for large doses of ADM (15 mg kg$^{-1}$) given immediately after irradiation (Moore & Broadbent, 1980). Phillips *et al.* (1979) have reported results very similar to the present ones with respect to the dependence on the time schedule of BLM and radiation treatment, but they failed to observe an increased $D_0$ for BLM 2 h before irradiation. However, the survival curve for the BLM-radiation combination was fitted to points embracing few crypt cells per circumference (half of the points below one crypt cell) which generally causes estimation of the $D_0$ to become inaccurate.

The pattern of the combined effects of MM-C and irradiation seen in Figure 4 may indicate that the two modalities act both in an additive way and by interference with repair mechanisms. The diminishing effect of MM-C when administered from a few hours before to 3 h after irradiation (Figure 4) may indicate a drug-induced decreased repair of sublethal radiation damage. The more pronounced effect on administration 6 h before irradiation may possibly be explained by radiation interference with repair of the effect of the drug. At present, there are no other data concerning the interactions of MM-C and irradiation in the intestinal tract epithelium. MM-C has also been shown to enhance the radiation induced skin reactions in mouse feet (von der Maase, 1984*b*).

The fact that *cis*-DDP enhanced the radiation response only when given before irradiation and decreased the $D_0$ for radiation alone may indicate a true radiosensitization. This hypothesis has previously been suggested in both *in vivo* and *in vitro* studies (Douple & Richmond, 1978, 1979 and 1982; Overgaard & Khan, 1981; Richmond & Powers, 1976; Richmond *et al.*, 1977). A selective enhancement at drug administration before irradiation was also found using the mouse foot skin scoring system (von der Maase, 1984*b*). Other studies on the interactions of *cis*-DDP and irradiation in the intestinal mucosa have confirmed *cis*-DDP to have the most pronounced effect by administration before irradiation (Burholt *et al.*, 1979; Luk *et al.*, 1979; Schenken *et al.*, 1979). However, these studies also reported some degree of enhancement by administration after irradiation and suggested that the effect of *cis*-DDP was due to a reduced repair of radiation injury.

As for the previously investigated combinations of drugs and irradiation (von der Maase, 1984*a*), the present study illustrates the complexity and severity of drug-radiation interactions in the intestinal tract epithelium. These observations should serve to warn clinicians and should be taken into consideration in the planning of combined drug-radiation treatments of patients. On the basis of the crypt survival data it seems that short intervals between the two treatment modalities yield the most serious injuries which for some combinations were found to be dramatic. Separation of drug and irradiation by days may effectively spare the tissue and administration of drugs before irradiation is likely to be more damaging than administration after radiation treatment. The results have, however, been based on single-dose experiments and fractionated experiments are obviously required to substantiate our knowledge and understanding of clinically relevant drug-radiation interactions. It is also important to study late effects of the combined treatments and much work therefore remains to be done in this area.

I am most grateful to I.M. Johansen for her excellent technical help, to Dr. J. Overgaard for his support throughout the study, to M. Juhl and P. Schjerbeck for preparing the histological sections, to L. Wagner for secretarial assistance and to M. Pilegaard for correcting the manuscript.

Adriamycin was kindly supplied by Farmitalia, Carlo Erba, bleomycin by H. Lundbeck & Co. A/S and mitomycin C and *cis*-platinum by Bristol-Myers A/S.

The present study was supported by the Danish Cancer Society, grants No. 24/79 and 90/80.

**References**

BURHOLT, D.R., HAGEMANN, R.F., COOPER, J.W., SCHENKEN, L.L. & LESHER, S. (1975). Damage and recovery assessment of the mouse jejunum to abdominal X-ray and adriamycin treatment. *Br. J. Radiol.*, **48**, 908.

BURHOLT, D.R., HAGEMANN, R.F., SCHENKEN, L.L. & LESHER, S. (1977). Influence of adriamycin and adriamycin-radiation combination on jejunal proliferation in the mouse. *Cancer Res.*, **31**, 22.

BURHOLT, D.R., SCHENKEN, L.L., KOVACS, C.J. & HAGEMANN, R.F. (1979). Response of the murine gastrointestinal epithelium to cis-dichlorodiammine-platinum II: radiation combinations. *Int. J. Radiat. Oncol. Biol. Phys.*, **5**, 1377.

DETHLEFSEN, L.A. & RILEY, R.M. (1979). The effects of adriamycin and X-irradiation on the murine duodenum. *Int. J. Radiat. Oncol. Biol. Phys.*, **5**, 507.

DOUPLE, E.B. & RICHMOND, R.C. (1978). Platinum complexes as radiosensitizers of hypoxic mammalian cells. *Br. J. Cancer*, **37** (Suppl. III), 98.

DOUPLE, E.B. & RICHMOND, R.C. (1979). Radiosensitization of hypoxic tumor cells by cis- and trans-dichlorodiammeineplatinum (II). *Int. J. Radiat. Oncol. Biol. Phys.*, **5**, 1369.

DOUPLE, E.B. & RICHMOND, R.C. (1982). Enhancement of the potentiation of radiotherapy by platinum drugs in a mouse tumor. *Int. J. Radiat. Oncol. Biol. Phys.*, **8**, 501.

LUK, K.H., ROSS, G.Y., PHILLIPS, T.L. & GOLDSTEIN, L.S. (1979). The interaction of radiation and cis-diammine-dichloroplatinum(II) in intestinal crypt cells. *Int. J. Radiat. Oncol. Biol. Phys.*, **5**, 1417.

MOORE, J. V. & BROADBENT, D. A. (1980). Survival of intestinal crypts after treatment by adriamycin alone or with radiation. *Br. J. Cancer*, **42**, 692.

MUGGIA, F.M., CORTES-FUNES, H. & WASSERMAN, T.H. (1978). Radiotherapy and chemotherapy in combined clinical trials: problems and promise. *Int. J. Radiat. Oncol. Biol. Phys.*, **4**, 161.

OVERGAARD, J. & KHAN, A.R. (1981). Selective enhancement of radiation response in a C3H mammary carcinoma by cisplatin. *Cancer Treat. Rep.*, **65**, 501.

PECKHAM, M.J. & COLLIS, C.H. (1981). Clinical objectives and normal tissue responses in combined chemotherapy and radiotherapy. *Bull. Cancer* (Paris), **68**, 132.

PHILLIPS, T.L. (1980). Clinical and experimental alterations in the radiation therapeutic ratio caused by cytotoxic chemotherapy. In *Radiation Biology in Cancer Research*, p. 567. (Ed. Meyn & Withers). Raven Press, New York.

PHILLIPS, T.L. & FU, K.K. (1976). Quantification of combined radiation therapy and chemotherapy effects on critical normal tissues. *Cancer*, **37**, 1186.

PHILLIPS, T.L., ROSS, G.Y., GOLDSTEIN, L.S. & BEGG, A.C. (1979). The interaction of radiation and bleomycin in intestinal crypt cells. *Int. J. Radiat. Oncol. Biol. Phys.*, **5**, 1509.

RICHMOND, R.C. & POWERS, E.L. (1976). Radiation sensitization of bacterial spores by cis-dichloro-diammineplatinum(II). *Radiat. Res.*, **68**, 251.

RICHMOND, R.C., ZIMBRICK, J.D. & HYKES, D.L. (1977). Radiation-induced DNA damage and lethality in E. coli as modified by the antitumour agent cis-dichloro-diammineplatinum(II). *Radiat. Res.*, **71**, 447.

ROSS, G.Y., PHILLIPS, T.L. & GOLDSTEIN, L.S. (1979). The interaction of irradiation and adriamycin in intestinal crypt cells. *Int. J. Radiat. Oncol. Biol. Phys.*, **5**, 1313.

SCHENKEN, L.L., BURHOLT, D.R., HAGEMANN, R.F. & KOVACS, C.J. (1979). Combined modality oncotherapies. Cell kinetic approaches for avoidance of gastrointestinal toxicity. *Front. Radiat. Ther. Oncol.*, **13**, 82.

SCHENKEN, L.L., BURHOLT, D.R., HAGEMANN, R.F. & LESHER, S. (1976). The modification of gastrointestinal tolerance and responses to abdominal irradiation by chemotherapeutic agents. *Radiology*, **120**, 417.

VON DER MAASE, H. (1984*a*). Interactions of radiation and 5-fluorouracil, cyclophosphamide or methotrexate in intestinal crypt cells. *Int. J. Radiat. Oncol. Biol. Phys.*, **10**, 77.

VON DER MAASE, H. (1984*b*). Effect of cancer chemotherapeutic drugs on the radiation-induced skin reactions in mouse feet. *Br. J. Radiol.* (in press).

VON DER MAASE, H. & OVERGAARD, J. (1983). Microcolony survival assay for jejunal crypt cells exposed to radiation alone and combined with cancer chemotherapeutic agents – methodological problems. *Int. J. Radiat. Biol.*, **43**, 45.

WITHERS, H.R. & ELKIND, M.M. (1970). Microcolony survival assay for cells of mouse intestinal mucosa exposed to radiation. *Int. J. Radiat. Biol.*, **17**, 261.